# Investigating Recovery After Subarachnoid Hemorrhage With the Imaging, Cognition and Outcome of Neuropsychological Functioning After Subarachnoid Hemorrhage (ICONS) Study: Protocol for a Longitudinal, Prospective Cohort Study

**DOI:** 10.2196/38190

**Published:** 2022-09-29

**Authors:** Sara Khosdelazad, Lieke S Jorna, Rob J M Groen, Sandra E Rakers, Marieke E Timmerman, Ronald J H Borra, Anouk van der Hoorn, Jacoba M Spikman, Anne M Buunk

**Affiliations:** 1 Neuropsychology Unit, Department of Neurology University Medical Centre Groningen University of Groningen Groningen Netherlands; 2 Department of Neurosurgery University Medical Centre Groningen University of Groningen Groningen Netherlands; 3 Department of Psychometrics and Statistics University of Groningen Groningen Netherlands; 4 Department of Radiology University Medical Centre Groningen University of Groningen Groningen Netherlands

**Keywords:** subarachnoid haemorrhage, cognition, neuroimaging, emotion and behavior, neuropsychology, longitudinal study, protocol, cohort study, bleeding, rupture, aneurysm, emotional, behavioral, brain damage

## Abstract

**Background:**

A subarachnoid hemorrhage is a hemorrhage in the subarachnoid space that is often caused by the rupture of an aneurysm. Patients who survive a subarachnoid hemorrhage have a high risk of complications and a negative long-term outcome.

**Objective:**

The aim of the Imaging, Cognition and Outcome of Neuropsychological functioning after Subarachnoid hemorrhage (ICONS) study is to investigate whether and to what extent deficits exist in multiple domains after subarachnoid hemorrhage, including cognition, emotion and behavior, and to investigate whether brain damage can be detected in patients with subarachnoid hemorrhage. We aim to determine which early measures of cognition, emotion and behavior, and brain damage in the subacute stage play a role in long-term recovery after subarachnoid hemorrhage. Recovery is defined as functioning at a societal participation level, with a focus on resuming and maintaining work, leisure activities, and social relationships over the long term.

**Methods:**

The ICONS study is an observational, prospective, single-center cohort study. The study includes patients with subarachnoid hemorrhage admitted to the Neurosurgery Unit of the University Medical Centre Groningen in the Netherlands. The inclusion criteria include diagnosis of an aneurysmal subarachnoid hemorrhage or an angiographically negative subarachnoid hemorrhage, sufficient ability in the Dutch language, and age older than 18 years. Patients will undergo neuropsychological assessment and magnetic resonance imaging 6 months after the subarachnoid hemorrhage. Furthermore, patients will be asked to fill in questionnaires on multiple psychosocial measures and undergo a structured interview at 6 months, 1 year, and 2 years after the subarachnoid hemorrhage. The primary outcome measure of the ICONS study is societal participation 1 year after the subarachnoid hemorrhage, measured with the Dutch version of the Impact on Participation and Autonomy questionnaire.

**Results:**

The study was launched in December 2019 and recruitment is expected to continue until June 2023. At the time of the acceptance of this paper, 76 patients and 69 healthy controls have been included. The first results are expected in early 2023.

**Conclusions:**

The ICONS study is the first to collect and combine data after subarachnoid hemorrhage in a variety of domains, including cognition, emotion and behavior, and brain damage. The results will contribute to a more comprehensive understanding of the consequences of both aneurysmal subarachnoid hemorrhage and angiographically negative subarachnoid hemorrhage, which may ultimately optimize timely treatment for this patient group by setting realistic and attainable goals to improve daily functioning.

**Trial Registration:**

Netherlands Trial Register NL7803; https://trialsearch.who.int/Trial2.aspx?TrialID=NL7803

**International Registered Report Identifier (IRRID):**

DERR1-10.2196/38190

## Introduction

### Background

A subarachnoid hemorrhage (SAH) is a hemorrhage in the subarachnoid space, which is located between the pia mater and the arachnoid mater. In 75% to 80% of cases, a SAH is caused by a ruptured intracranial aneurysm; this is termed an aneurysmal SAH (aSAH). In approximately 15% of cases, no structural cause for the hemorrhage can be detected; this is defined as an angiographically negative SAH (anSAH). Despite the fact that SAH only accounts for 3% to 5% of all strokes, it has the highest morbidity and mortality rates of all stroke types [1]. One of the main clinical characteristics of SAH is an extremely severe headache with an acute onset. Half of all patients experience a loss of consciousness at onset [2]. Other symptoms include nausea, vomiting, neck stiffness, and focal neurological deficits. The main complications that may occur are acute hydrocephalus, recurrent hemorrhage, and cerebral vasospasm [3].

A distinctive feature of SAH is the relatively young age at which it occurs, with a peak incidence between 40 and 60 years [4]. Although recovery of functional independence is common, many SAH patients still experience negative long-term effects in everyday life functioning, which has a large impact on both patients and relatives. For example, previous studies have shown that more than 50% of all SAH patients are unable to resume their previous work, even in the absence of physical limitations [5,6]. In addition, many patients experience problems in leisure activities and social contacts after SAH [7,8]. Factors such as cognitive complaints, mood disorders, and behavioral deficits are related to these problems.

### Cognitive and Emotional/Behavioral Consequences

After aSAH, disorders arise in both basic cognitive functions (such as memory, attention, and language) and higher-order functions (such as executive functions and social cognition) [5,9]. Even patients who are functionally independent (ie, able to perform activities of daily living without help) can experience cognitive impairment, causing disability in patients for months or even years after aSAH [7,10]. In addition, high rates of residual symptoms have been reported after aSAH, predominantly including fatigue, anxiety, and mood disturbances. These persistent cognitive and emotional sequelae may have a significant impact on the social and work life of patients. Previous studies have found that approximately 40% of aSAH patients are not able to return to their previous work between 1 to 4 years after ictus [6,11-13]. This can lead to reduced quality of life (QoL) in this patient group [14,15].

Cognitive outcomes following anSAH have been less well studied, and the existing literature shows contradictory results. While some studies have not found evidence of cognitive deficits in this patient group, others have found lower scores for divided attention, information processing speed, and memory [16-18]. A recent systematic review by Burke and colleagues [19] implied that a diffuse pattern of cognitive impairments can be seen in anSAH patients, with primarily attention and executive functioning being affected. Moreover, subjective complaints related to cognitive functioning, fatigue, and mood have been steadily reported after anSAH and seem to significantly influence QoL [16,20-22].

Furthermore, both aSAH and anSAH patients show changes in behavior and personality, such as apathy [23] and inadequate social behavior [24-26]. Changes in behavior are often accompanied by impaired self-awareness [27]. This casts doubt on whether self-reported measures alone can be used to assess the presence and frequency of long-term consequences of SAH and emphasizes the importance of obtaining measures from informants. Although cognitive, emotional, and behavioral deficits after SAH have been frequently explored, the explanatory value of these domains for long-term recovery in the subacute stage after SAH has not yet been investigated.

### Brain Damage After SAH

SAH may result in diffuse injury caused by the initial hemorrhage, complications (eg, vasospasms, hydrocephalus, and recurrent hemorrhage), or secondary damage due to delayed cerebral ischemia. Treatment of the aneurysm in the case of aSAH generally involves either microsurgical occlusion (clipping) or endovascular obliteration (coiling, stenting, or both). Previous studies have investigated the effects of clinical parameters (eg, the characteristics of the aneurysm, the treatment modality, vasospasm, and cerebrospinal fluid drainage) on outcomes after SAH. However, no clear evidence for the effects of these parameters has been found. For example, in a study of 32 aSAH patients, no association was found between the site of the aneurysm or the mode of treatment with neuropsychological outcomes [28]. The authors did find that the severity of the hemorrhage was significantly linked with intelligence and memory. Another study, which included 23 aSAH patients who underwent coiling or clipping, found no differences in cognitive outcomes with different treatment modalities [29]. The aneurysm location also did not seem to be related to the resumption of social activities in a group of 200 aSAH patients [7]. It is therefore of great importance to take a more in-depth look into the diffuse brain damage that may occur after SAH.

Brain imaging techniques, such as magnetic resonance imaging (MRI), can identify structural changes after SAH. For example, T2-weighted, fluid-attenuated inversion recovery and 3D T1-weighted images can depict a variety of etiologies, such as ischemic lesions and brain abnormalities that can be the result of aneurysm treatment (either microsurgical or endovascular), lesions associated with external ventricular drains or permanent shunt placement, and previous infarctions [30]. A recent study obtained complete T1-weighted data 3 months post-aSAH and found no difference in total gray-matter or white-matter volume between patients and controls [31]. However, that study did find a significantly higher gray-matter volume in the cerebellum of aSAH patients than controls, and the study also found an association with neurocognitive impairment.

There are some promising imaging techniques that have not been used, or have barely been used, in the SAH patient population; these techniques have the potential to contribute to knowledge about the long-term cognitive consequences of SAH. First, diffusion tensor imaging (DTI) can identify microstructural white matter lesions that are not visible with standard brain MRI [32]. The most-examined parameters in DTI studies are fractional anisotropy (FA), mean diffusivity (MD), and apparent diffusion coefficient (ADC). Previous research has shown that 2 weeks after ictus, aSAH patients have higher MD values than patients with an unruptured intracranial aneurysm [33]. Higher MD values were associated with cognitive impairment 3 months after ictus in these patients. Another study found decreased FA in the corpus callosum and increased ADC values in the frontal centrum semiovale in aSAH patients 8 to 10 days after ictus [34]. To date, DTI has not been used in anSAH patients, and it is therefore unknown to what extent damage in the white-matter microstructure relates to less favorable cognitive outcomes in this patient group and whether this differs between aSAH and anSAH patients. A more advanced and promising technique is diffusion kurtosis imaging (DKI). DKI has the potential to further delineate microstructural aspects of the white matter [35] and has been used in the assessment and differentiation of stroke, brain tumor, and neurodegenerative diseases [36,37]. To our knowledge, DKI has not yet been used in SAH patients. Second, arterial spin labeling data can be used to measure blood flow in brain tissue (ie, perfusion) and has demonstrated clinical usefulness in various diseases, including vascular spasm, brain tumors, and Alzheimer disease [38-40]. Third, susceptibility weighted imaging (SWI), a relatively new tool for imaging blood vessels, hemorrhage, iron and blood products, and calcifications [41,42], has been used to detect parenchymal hemorrhage and can detect small amounts of SAH more sensitively than conventional gradient-echo sequences [43]. SWI data can be used as input for quantitative susceptibility mapping (QSM.) QSM is a novel MRI technique that has been used to study brain iron and blood degeneration products in various patient populations, such as those with traumatic brain injury, brain tumors, or neurodegenerative diseases [44]. QSM has the potential to indicate hemorrhages and microhemorrhages in a more accurate and qualitative way than SWI in a variety of neuropathologies [45]. Fourth, vessel architectural imaging (VAI) is a new dual-echo dynamic susceptibility contrast (DSC) perfusion sequence technique that can reveal the microvascular architecture and oxygen saturation status. To date, VAI has only been used in tumor patients and has been shown to provide better insights than other noninvasive imaging techniques into the complex nature and heterogeneity of vascular changes [46,47]. In addition, single-echo DSC can be used to gain a better understanding of heterogeneity in microvascular and capillary transit time and the oxygen extraction fraction [48]. Finally, synthetic MRI is a technique whereby a single scan can be used to create multiple different contrast-weighted snapshots, allowing automatic brain segmentation and myelin volumetric measurements [49]. In short, the above MRI methods may allow the evaluation of brain damage after SAH, particularly vascular damage, in a more comprehensive and sensitive way. Moreover, the application of these techniques may offer important new insights into the brain damage that underlies cognitive impairment and difficulties in daily functioning after SAH.

### Objectives

The overall aims of the Imaging, Cognition and Outcome of Neuropsychological functioning after Subarachnoid hemorrhage (ICONS) study are to determine whether and to what extent there are deficits in multiple domains after SAH (including cognition, emotion, and behavior) and to investigate whether brain damage can be detected in this patient group. If this is the case, we aim to determine which early measures (including measures of cognition, emotion and behavior, and brain damage) in the subacute stage play a role in long-term recovery after SAH. Recovery is defined as societal participation, focusing on resuming and maintaining work, leisure activities, and social relationships over the long term. Furthermore, the study has 12 specific objectives that focus on the domains of cognition, emotion and behavior, and brain damage, which will be targeted in multiple future papers. These objectives are described in Textbox 1. We expect that all studied measures will have an effect on recovery individually, but that in an overall explanatory model the neuropsychological factors will play a more decisive role than the initial brain damage. Early identification of decisive factors may provide target points for rehabilitation in this patient group.

**Objectives of the Imaging, Cognition and Outcome of Neuropsychological Functioning After Subarachnoid Hemorrhage (ICONS) study.**

**Overall aims**

We aim to determine whether and to what extent deficits exist after subarachnoid hemorrhage in multiple domains, including cognition, emotion, and behavior, and to investigate whether brain damage can be detected in patients with subarachnoid hemorrhage. We also aim to determine which early measures of cognition, emotion and behavior, and brain damage in the subacute stage play a role in long-term recovery 1 year after subarachnoid hemorrhage.

**Aims in the cognition domain**

1. Determine whether and to what extent there are deficits in various cognitive domains (eg, executive functioning, language, memory, processing speed, social cognition, and visuoconstructive skills) 6 months after subarachnoid hemorrhage
2. Investigate whether and to what extent there are relationships between cognitive functioning 6 months after subarachnoid hemorrhage and individual patient characteristics (eg, age, treatment, type of subarachnoid hemorrhage, severity of subarachnoid hemorrhage, and complications)
3. Investigate whether and to what extent there are relationships between cognitive functioning 6 months after subarachnoid hemorrhage and emotional and behavioral characteristics (eg, anxiety, mood complaints, fatigue, and posttraumatic stress) 1 and 2 years after subarachnoid hemorrhage
4. Investigate the relationship between cognitive functioning 6 months after subarachnoid hemorrhage and recovery 1 and 2 years after subarachnoid hemorrhage

**Aims in the emotion and behavior domain**

1. Determine emotional and behavioral consequences in various domains (eg, anxiety, mood complaints, fatigue, subjective cognitive complaints, posttraumatic stress, and quality of life) 6 months after subarachnoid hemorrhage
2. Investigate whether and to what extent there are relationships between emotional and behavioral consequences and individual patient characteristics (eg, age, treatment, type of subarachnoid hemorrhage, severity of subarachnoid hemorrhage, and complications)
3. Determine the improvement of emotional and behavioral consequences over time, as measured at 6 months, 1 year, and 2 years after subarachnoid hemorrhage
4. Investigate the relationship between emotional and behavioral consequences 6 months after subarachnoid hemorrhage and recovery 1 year and 2 years after subarachnoid hemorrhage

**Aims in the brain damage domain**

1. Determine differences in brain tissue between patients and matched healthy controls using advanced imaging techniques
2. Determine differences in brain tissue between patients with aneurysmal subarachnoid hemorrhage and angiographically negative subarachnoid hemorrhage using advanced imaging techniques
3. Investigate the relationship between impairments in cognitive domains (eg, executive functioning, language, memory, processing speed, social cognition, and visuoconstructive skills) and specific lesion patterns as identified with advanced imaging techniques
4. Investigate the relationship between brain damage 6 months after subarachnoid hemorrhage and recovery 1 year and 2 years after subarachnoid hemorrhage

## Methods

### Study Design

The ICONS study is a longitudinal, prospective, single-center cohort study that aims to include 150 patients with SAH who will be followed up at 6 months, 1 year, and 2 years after ictus.

### Study Population

Patients are being recruited from the Neurosurgery Unit of the University Medical Centre Groningen (UMCG), the Netherlands. Based on the admission rate of approximately 120 patients with SAH per year at the UMCG, and accounting for unwillingness to participate and loss to follow-up, we aim to recruit 150 patients with SAH. In addition, 100 matched healthy controls will be recruited through convenience sampling. Inclusion criteria are an aSAH or anSAH diagnosis established by means of a computed tomography (CT) scan. The presence or absence of an intracranial aneurysm (ie, aSAH or anSAH, respectively) is evaluated using CT angiography, digital subtraction angiography, or both. Other inclusion criteria include sufficient knowledge of the Dutch language and age older than 18 years. Exclusion criteria for both patients and healthy controls are a poor physical condition that makes it impossible for participants to undergo the neuropsychological assessment or MRI scan and a history of severe neurological disorders.

### Study Procedures

#### Patients

Data collection is administered and performed by 2 researchers (ie, neuropsychologists). The researchers inform patients about the study upon discharge from the hospital by handing them written information regarding the purpose and design of the study. Six weeks after SAH, patients have an outpatient check-up appointment with their treating physician. Following this appointment, patients have the opportunity to speak with the researchers and pose questions about the study. Written informed consent is then obtained. Patients who do not consent to the study receive standard clinical aftercare according to the UMCG SAH protocol.

After informed consent is obtained, demographic and medical data are obtained from patients’ medical records, including the World Federation of Neurological Surgeons (WFNS) grade. The WFNS grading scale is the most-used classification method for determining the initial clinical condition after SAH and is based on loss of consciousness; it ranges from 1 to 5 [50]. The researchers schedule the neuropsychological assessment (NPA) and MRI scan on 2 separate days, approximately 6 months post-SAH. The maximum time between the MRI and the NPA is 1 month. The MRI scan takes approximately 1 hour (a 50-minute scan time and 10-minute preparation time).

Patients receive questionnaires regarding emotion and behavior 2 weeks prior to the NPA (Table 1). These questionnaires are sent digitally via the secure web app Research Electronic Data Capture (REDCap) or, upon request, on paper. At the NPA appointment, the investigator scrutinizes the questionnaires (eg, checks for missing data or questions with multiple answers selected). The NPA takes approximately 3 hours and consists of a structured interview with the patient and a neuropsychological test battery covering different cognitive domains (Table 1). During the interview, the researchers document the patient’s level of education, their handedness, and their self-reported cognitive, emotional, and behavioral complaints. Furthermore, a questionnaire regarding the patient’s sleep patterns, resumption of work, and the type and amount of any previously received rehabilitation or psychological treatment is administered. We expect the duration of the NPA to be feasible, because a previously conducted study with SAH patients made use of a more-extensive neuropsychological test battery that was shown to be feasible for this patient group [11]. However, if needed, the patients are allowed small breaks during the NPA, and if necessary, the appointment can also be divided over 2 days. Patients receive follow-up assessments 1 year and 2 years post-SAH. For the 1-year follow-up, the researchers schedule an appointment with the patient at the UMCG. The 2-year follow-up assessment is a telephone interview. The patients receive the questionnaires 2 weeks before each follow-up assessment.

**Table 1:** Overview of the assessments used for the Imaging, Cognition and Outcome of Neuropsychological functioning after Subarachnoid hemorrhage (ICONS) study.

Assessments			Construct		Six-month follow up		One-year follow up		Two-year follow up
**General information (patients)**									
	Demographics	Age, sex, education		✓					
	Medical data	Treatment, SAH^a^ type, aneurysm, complications		✓					
	Modified Rankin Scale	Functional status		✓		✓		✓	
	Sleep-work-treatment questionnaire	Brain structural changes		✓		✓		✓	
**Neuropsychological tests (patients)**									
	Rey Complex Figure Test copy	Visuospatial construction		✓					
	15 Words Test	Verbal memory		✓					
	Digit Span Test of the Wechsler Adult Intelligence Scale IV	Memory span/working memory		✓					
	Letter Fluency test	Executive functioning		✓					
	Zoo Map Test of the Behavioural Assessment of the Dysexecutive Syndrome	Executive functioning		✓					
	Key Search Test of the Behavioural Assessment of the Dysexecutive Syndrome	Executive functioning		✓					
	Trail making test	Complex attention		✓					
	Vienna Test System reaction time test	Complex attention		✓					
	Semantic Fluency test	Language		✓					
	Hinting Task	Social cognition		✓					
	Cartoons test	Social cognition		✓					
	Facial expression of emotion: stimuli and tests	Social cognition		✓					
	Dutch Adult Reading Test	Premorbid intelligence		✓					
**Questionnaires (patients)**									
	Impact on Participation and Autonomy	Participation				✓		✓	
	Utrecht Scale for Evaluation of Rehabilitation	Participation activities		✓		✓		✓	
	Checklist for cognitive and emotional consequences following stroke	Cognitive and emotional complaints		✓		✓		✓	
	Dysexecutive questionnaire	Dysexecutive syndrome		✓		✓		✓	
	Dutch Multifactor Fatigue Scale	Fatigue		✓		✓		✓	
	Hospital Anxiety and Depression Scale	Anxiety and depression		✓		✓		✓	
	Life Satisfaction Questionnaire	Quality of life		✓		✓		✓	
	Impact of Events Scale	PTSD^b^		✓		✓		✓	
	Utrecht Coping List	Coping		✓					
**Questionnaires (proxies)**									
	Utrecht Scale for Evaluation of Rehabilitation	Participation activities		✓		✓		✓	
	Checklist for cognitive and emotional consequences following stroke	Cognitive and emotional complaints		✓		✓		✓	
	Dysexecutive questionnaire	Dysexecutive syndrome		✓		✓		✓	
	Socioemotional Dysfunction Scale	Social dysfunction		✓		✓		✓	
**MRI^c^ brain scanning (patients)**									
	3D-T1 MRI	Brain structural changes		✓					
	T2 MRI	Brain structural changes		✓					
	Fluid attenuated inversion recovery	Brain segmentation		✓					
	Synthetic MRI	Myelin volumetric measurements		✓					
	Diffusion kurtosis imaging/diffusion tensor imaging	Microstructural white matter lesions		✓					
	Arterial spin labeling	Perfusion parameters		✓					
	Vessel architectural imaging	Microvascular architecture		✓					
	Susceptibility weighted imaging/quantitative susceptibility mapping	(Micro)hemorrhagic damage		✓					

^a^SAH: subarachnoid hemorrhage.

^b^PTSD: posttraumatic stress disorder.

^c^MRI: magnetic resonance imaging.

#### Proxies

Patients are requested to bring a proxy (eg, a significant other or close relative) to the NPA. These proxies are asked to fill out several questionnaires (Table 1). Additionally, a checklist of cognitive and emotional consequences following stroke is filled out during a short individual interview. Informed consent is obtained from the proxy before study participation. Like the patients, they fill out online (or paper, upon request) questionnaires at the 1-year and 2-year follow-ups.

#### Healthy Controls

A total of 100 healthy controls will be included for the NPA, of which 30 will undergo an MRI scan. Subjects are first included for the NPA and afterwards asked whether they are also willing to undergo an MRI scan. The MRI protocol for the healthy controls is the same as for the patients. If an unexpected abnormality is found on the MRI scan, the general practitioner of the healthy control will be contacted. The healthy controls give permission for this procedure when signing the informed consent form. The test battery of the NPA is the same as for the patients, but the structured interview and proxy measures are excluded. The healthy control group will be matched for age, gender, and education using frequency matching.

### Data Collection Instruments

#### Impact on Participation and Autonomy

The primary outcome measure of the ICONS study will be the Dutch version of the Impact on Participation and Autonomy (IPA) [51] scale, assessed 1 year after the SAH via a structured interview. The IPA scale has been developed to assess problems experienced by patients regarding autonomy and participation. The questionnaire contains a total of 41 items, each scored on a 5-point rating scale with the following labels: 0 (very good), 1 (good), 2 (fair), 3 (poor), and 4 (very poor). The items are divided into 5 domains, of which the following 3 will be used in the ICONS study: family role (7 items), social relationships (7 items), and work and education (6 items). The total score is calculated by summing up these 3 domains and ranges from 0 to 80. Since these domains measure data at an ordinal level, the results will be dichotomized into “complete recovery of societal participation” (for scores from 0-20) and “incomplete recovery of societal participation” (for scores 21-80) to perform logistic regression analyses.

#### Neuropsychological Assessment

Table 1 lists the cognitive domains and the associated neuropsychological tests that will be used. All tests are commonly used in both research and clinical practice within the Netherlands and have either normative data or data from healthy controls available. To control for insufficient effort of participants, we will use embedded indicators, such as the 15 Words Test and the Digit Span Task. Performance of the patients will be compared with that of the healthy controls. Raw test results will be converted into relevant statistics (eg, *z* scores, *t* scores, or percentiles), by using normative scores for standard neuropsychological tests.

#### Questionnaires

Questionnaires regarding emotional and behavioral disturbances are administered 6 months, 1 year, and 2 years after SAH to investigate how patients recover over time (Table 1).

#### Magnetic Resonance Imaging

MRI data are acquired on a 3-tesla scanner (Magnetom Prisma, Siemens) with a standard 64-channel head coil. A standardized scanning protocol is used. The MRI sequences are listed in Table 1. The MRI data will be analyzed and processed with various software packages, such as the Functional Magnetic Resonance Imaging of the Brain Software Library (FSL) [52], the voxel-based morphometry (VBM) toolbox under statistical parametric mapping (Wellcome Department of Imaging Neuroscience), and the cNeuro image quantification Conformité Européenne-marked tool (Combinostics Ltd). The FSL is a comprehensive library of analysis tools for functional MRI and DTI brain imaging data. VBM is an automated technique for the analysis of neuroanatomical images that identifies regional differences in gray matter and white matter between groups of subjects without a prior region of interest. cNeuro provides fully automated brain MRI quantification. Perfusion data will be analyzed using a research version of Nordic (NordicNeuroLab) and a research version of Cercare Medical Neurosuite (Cercare Medical).

### Data Management

The data are stored in an electronic case report form (eCRF) using REDCap. Imaging data are stored on a local secured server at the UMCG. Metadata are stored in the eCRF. Study monitoring is performed by in-house study monitors from the UMCG. Since our data contain potentially sensitive information and therefore pose privacy concerns (even when anonymized), there will be ethical restrictions on sharing our data set. Restrictions will be imposed by the Medical Ethical Committee of the UMCG.

### Statistical Analysis

Descriptive statistics (including frequencies, means, and 95% CIs) will be used to describe the samples, and data from each variable will be summarized separately for patients and healthy controls. Deficits in multiple domains will be determined by comparing patients with healthy controls (at the group level) and by comparing patients’ scores with normative data (at the individual level). Test performance below the 10th percentile will be considered impaired. Statistical analyses will be performed according to the applicable objectives shown in Textbox 1. Furthermore, we will develop three explanatory models: (1) cognition, (2) emotion and behavior, and (3) brain damage; this will use binary logistic regression analyses with recovery (ie, IPA ≤20 or IPA ≥21) as the dependent variable. A significance of α=.05 will be used as the cut-off for each variable. Only variables that are expected to have a strong relationship with the dependent variable will be included, based on a priori expectations from the literature and clinical experience. Measures for the same cognitive domains (Table 1) will be taken together as a composite score. The composite score will be generated by converting raw test scores from individual measures to *z* scores and averaging them. Lastly, we will create an overall comprehensive explanatory model that takes into account the most sensitive measures from each domain (ie, cognition, emotion and behavior, and brain damage) to see how these measures relate to each other in explaining recovery 1 year after SAH.

### Sample Size

In multivariable logistic regression models, a sample size of 10 events per variable (EPVs) is desirable to preserve the validity of the model [53]. This rule indicates that 1 independent variable can be studied for every 10 events. For logistic regression, the number of events is given by the smallest number of events within the 2 outcome categories. Based on previous literature, we expect that around half the patients (n=75) will have a complete recovery 1 year after SAH, while the other half (n=75) will have an incomplete recovery 1 year after SAH [7]. This implies that 7 (ie, 75 / 10 = 7.5) variables will be able to be reliably fitted to the model. However, in analyses of causal influences in observational data, control for confounding variables may require adjustment for more covariates than the rule of 10 EPVs allows. Vittinghof and McCullough [54] demonstrated that problems are fairly frequent with 2 to 4 EPVs, uncommon with 5 to 9 EPVs, and still observed with 10 to 16 EPVs when regarding CI coverage less than 93 percent, type 1 error larger than 7 percent, or relative bias larger than 15 percent as problematic. Following these findings, using 5 EPVs will allow for 15 (ie, 75 / 5 = 15) independent variables in each model.

Additionally, 100 healthy controls will be included in order to perform comparisons with the patient group and determine reference values. Since the ICONS study will use new neuroimaging methods, reference values will be needed to compare and interpret the MRI data. Healthy subjects show less variability in results than patients, which means that a smaller number of healthy subjects will be required. Previous MRI studies with SAH patients have shown that the number of healthy controls targeted in the ICONS study will be sufficient [55,56].

### Ethics Approval and Consent to Participate

The ICONS study will be conducted according to the principles of the Declaration of Helsinki (World Medical Association Declaration of Helsinki, 64th World Medical Association General Assembly, Fortaleza, Brazil, October 2013) and the national and international standards of Good Clinical Practice. Potential participants receive detailed written and oral information on the study procedures and all participants will provide written informed consent. Ethical approval of the study protocol was obtained from the Medical Ethical Committee of the UMCG (2019/346). The ICONS study protocol is registered at the Dutch Central Committee of Research Involving Human Subjects, with trial registration number NL69873.042.19. In addition, the study was registered on June 19th, 2019, at the Netherlands Trial Register with identifier NL7803.

## Results

**Figure 1:**
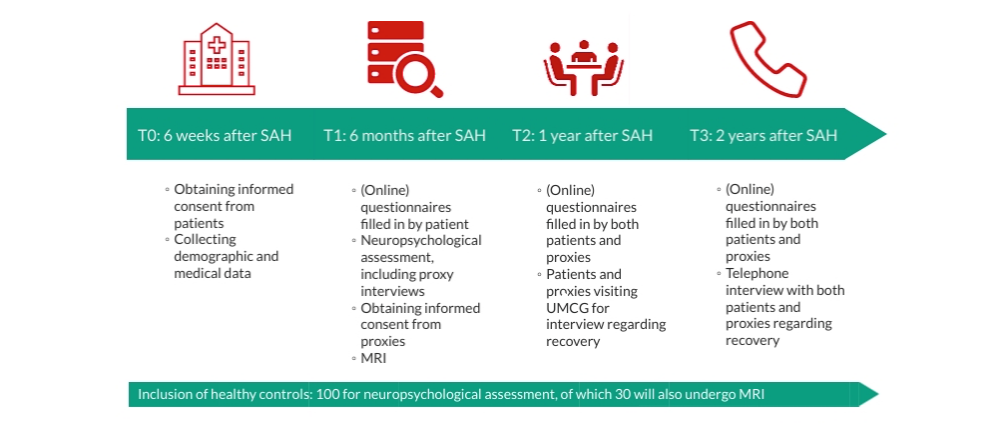
Timetable of the Imaging, Cognition and Outcome of Neuropsychological functioning after Subarachnoid hemorrhage (ICONS) study. MRI: magnetic resonance imaging; SAH: subarachnoid hemorrhage; UMCG: University Medical Centre Groningen.

As of December 2019, we enrolled 76 patients and 69 healthy controls. Recruitment is planned to continue until June 2023. The first publications are expected in early 2023. An overview of the timeline and assessments of the ICONS study is depicted in Figure 1.

## Discussion

The ICONS study is the first to combine the domains of cognition, emotions and behavior, as well as the first to use MRI imaging biomarkers, to investigate recovery in a longitudinal cohort of SAH patients and to determine which domain plays the most decisive role in long-term recovery, and consequently find better targets for rehabilitation. We believe this study will add to the existing literature for several reasons.

First, the most commonly used scales to determine functional outcomes in SAH patients are the modified Rankin Scale (mRS) and the Glasgow Outcome Scale (GOS). However, previous research has shown that patients with a “good” outcome (ie, a GOS score of 1) may still have substantial cognitive impairments and emotional problems, which indicates that these scales are not sufficiently sensitive [57,58]. Additionally, a study of 214 aSAH patients showed that one-third of patients with an mRS score of 0 (ie, “no symptoms at all”) were unable to return to work, and that 1 in 6 experienced mood disturbances [59]. The ICONS study measures outcome as functioning at a participation level, with a focus on both resuming and maintaining work, leisure activities, and social activities over the long term. This is especially important for patients with SAH, because SAH often occurs at a relatively young age, when patients are in a stage of life in which they generally have important tasks and responsibilities in their family, work, and social environment.

Second, whereas the vast majority of SAH research only focuses on aSAH patients, the current study also includes anSAH patients. In comparison to aSAH patients, clinical outcomes for anSAH patients are significantly better, including lower rates of delayed cerebral ischemia and radiologic infarction [60]. Therefore, anSAH is often considered a benign disorder. However, diffuse cognitive deficits have been demonstrated [19], and subjective complaints regarding cognition, fatigue, and mood are common [16,21,22]. The ICONS study will, furthermore, be the first to perform MRI imaging in anSAH patients. This will provide more knowledge into whether brain damage is the underlying cause of the problems experienced by this patient group.

Third, this is the first study to assess a variety of new and advanced neuroimaging methods in SAH patients. These techniques are more sensitive than conventional MRI (eg, for detecting cerebral microbleeds and vasospasm and their consequences in the brain). Their application may therefore offer important new insights into the brain damage underlying the cognitive disorders and difficulties in daily functioning after SAH.

A limitation of the study design is that only those patients who are able to undergo the NPA and MRI scans are included. Therefore, the results may not be applicable to more severely impaired SAH patients. Additionally, this is a single-center study, which might also affect the generalizability of the results.

In conclusion, the results of the ICONS study will contribute to a more comprehensive and timely understanding of the consequences of both aSAH and anSAH, which may ultimately allow the optimization of timely treatment for this patient group by setting realistic and attainable goals to improve daily functioning. Furthermore, based on the findings of this study, psychoeducation for patients and their families might be improved.

## Abbreviations

ADC: apparent diffusion coefficient
anSAH: angiographically negative subarachnoid hemorrhage
aSAH: aneurysmal subarachnoid hemorrhage
CT: computed tomography
DKI: diffusion kurtosis imaging
DSC: dynamic susceptibility contrast
DTI: diffusion tensor imaging
eCRF: electronic case report form
EPV: event per variable
FA: fractional anisotropy
FSL: FMRIB Software Library
GOS: Glasgow Outcome Scale
ICONS: Imaging, Cognition and Outcome of Neuropsychological functioning after Subarachnoid hemorrhage
IPA: Impact on Participation and Outcome
MD: mean diffusivity
MRI: magnetic resonance imaging
mRS: modified Rankin scale
NPA: neuropsychological assessment
QoL: quality of life
QSM: quantitative susceptibility mapping
SAH: subarachnoid hemorrhage
SWI: susceptibility weighted imaging
UMCG: University Medical Centre Groningen
VAI: vessel architectural imaging
VBM: voxel-based morphometry
WFNS: World Federation of Neurological Surgeons

## Data Availability

Depending on the type of data and associated privacy regulations, data from the Imaging Cognition and Outcome of Neuropsychological Functioning After Subarachnoid Hemorrhage (ICONS ) study will be made available upon reasonable request to the principal investigator (AMB).

## References

[1] Feigin VL, Lawes CM, Bennett DA, Barker-Collo SL, Parag V (2009). Worldwide stroke incidence and early case fatality reported in 56 population-based studies: a systematic review. Lancet Neurol.

[2] Hop JW, Rinkel GJE, Algra A, van Gijn J (1999). Initial loss of consciousness and risk of delayed cerebral ischemia after aneurysmal subarachnoid hemorrhage. Stroke.

[3] van Gijn J, Kerr RS, Rinkel GJ (2007). Subarachnoid haemorrhage. Lancet.

[4] Bederson JB, Connolly ES, Batjer HH, Dacey RG, Dion JE, Diringer MN, Duldner JE, Harbaugh RE, Patel AB, Rosenwasser RH, American Heart Association (2009). Guidelines for the management of aneurysmal subarachnoid hemorrhage: a statement for healthcare professionals from a special writing group of the Stroke Council, American Heart Association. Stroke.

[5] Al-Khindi Timour, Macdonald R Loch, Schweizer Tom A (2010). Cognitive and functional outcome after aneurysmal subarachnoid hemorrhage. Stroke.

[6] Passier PE, Visser-Meily JM, Rinkel GJ, Lindeman E, Post MW (2011). Life satisfaction and return to work after aneurysmal subarachnoid hemorrhage. J Stroke Cerebrovasc Dis.

[7] Buunk AM, Groen RJM, Veenstra WS, Spikman JM (2015). Leisure and social participation in patients 4-10 years after aneurysmal subarachnoid haemorrhage. Brain Inj.

[8] Carter BS, Buckley D, Ferraro R, Rordorf G, Ogilvy CS (2000). Factors associated with reintegration to normal living after subarachnoid hemorrhage. Neurosurgery.

[9] Rinkel GJ, Algra A (2011). Long-term outcomes of patients with aneurysmal subarachnoid haemorrhage. Lancet Neurol.

[10] Wong GKC, Lam SW, Ngai K, Wong A, Siu D, Poon WS, Mok V, Cognitive Dysfunction after Aneurysmal Subarachnoid Hemorrhage Investigators (2013). Cognitive domain deficits in patients with aneurysmal subarachnoid haemorrhage at 1 year. J Neurol Neurosurg Psychiatry.

[11] Buunk AM, Spikman JM, Metzemaekers JDM, van Dijk JMC, Groen RJM (2019). Return to work after subarachnoid hemorrhage: The influence of cognitive deficits. PLoS One.

[12] Turi ER, Conley Y, Crago E, Sherwood P, Poloyac SM, Ren D, Stanfill AG (2019). Psychosocial comorbidities related to return to work rates following aneurysmal subarachnoid hemorrhage. J Occup Rehabil.

[13] Hütter B O, Kreitschmann-Andermahr I, Gilsbach J M (2001). Health-related quality of life after aneurysmal subarachnoid hemorrhage: impacts of bleeding severity, computerized tomography findings, surgery, vasospasm, and neurological grade. J Neurosurg.

[14] Kreiter KT, Copeland D, Bernardini GL, Bates JE, Peery S, Claassen J, Du YE, Stern Y, Connolly ES, Mayer SA (2002). Predictors of cognitive dysfunction after subarachnoid hemorrhage. Stroke.

[15] Sonesson B, Kronvall Erik, Säveland Hans, Brandt Lennart, Nilsson Ola G (2018). Long-term reintegration and quality of life in patients with subarachnoid hemorrhage and a good neurological outcome: findings after more than 20 years. J Neurosurg.

[16] Boerboom W, Heijenbrok-Kal M, Khajeh L, van Kooten Fop, Ribbers G (2014). Differences in cognitive and emotional outcomes between patients with perimesencephalic and aneurysmal subarachnoid haemorrhage. J Rehabil Med.

[17] Buunk AM, Groen RJM, Veenstra WS, Metzemaekers JDM, van der Hoeven JH, van Dijk JMC, Spikman JM (2016). Cognitive deficits after aneurysmal and angiographically negative subarachnoid hemorrhage: Memory, attention, executive functioning, and emotion recognition. Neuropsychology.

[18] Krajewski K, Dombek S, Martens T, Köppen Johannes, Westphal M, Regelsberger J (2014). Neuropsychological assessments in patients with aneurysmal subarachnoid hemorrhage, perimesencephalic SAH, and incidental aneurysms. Neurosurg Rev.

[19] Burke T, Hughes S, Carr A, Javadpour M, Pender N (2018). A systematic review of cognitive outcomes in angiographically negative subarachnoid haemorrhage. Neuropsychol Rev.

[20] Beseoglu K, Pannes S, Steiger HJ, Hänggi Daniel (2010). Long-term outcome and quality of life after nonaneurysmal subarachnoid hemorrhage. Acta Neurochir (Wien).

[21] Madureira S, Canhão P, Guerreiro M, Ferro JM (2000). Cognitive and emotional consequences of perimesencephalic subarachnoid hemorrhage. J Neurol.

[22] McIntosh A, Thomas Ajith (2015). Health-related quality-of-life outcomes: comparing patients with aneurysmal and nonaneurysmal subarachnoid hemorrhage. J Neurosci Nurs.

[23] Tang WK, Wang L, Tsoi KK, Yasuno F, Kim JS (2022). Apathy after subarachnoid haemorrhage: A systematic review. J Psychosom Res.

[24] Ogden J, Utley T, Mee E (1997). Neurological and psychosocial outcome 4 to 7 years after subarachnoid hemorrhage. Neurosurgery.

[25] Storey P (1972). Emotional disturbances before and after subarachnoid haemorrhage. Ciba Found Symp.

[26] Buunk AM, Spikman JM, Veenstra WS, van Laar PJ, Metzemaekers JD, van Dijk JMC, Meiners LC, Groen RJ (2017). Social cognition impairments after aneurysmal subarachnoid haemorrhage: Associations with deficits in interpersonal behaviour, apathy, and impaired self-awareness. Neuropsychologia.

[27] Buchanan KM, Elias LJ, Goplen GB (2000). Differing perspectives on outcome after subarachnoid hemorrhage: the patient, the relative, the neurosurgeon. Neurosurgery.

[28] Haug Tonje, Sorteberg Angelika, Sorteberg Wilhelm, Lindegaard Karl-Fredrik, Lundar Tryggve, Finset Arnstein (2007). Cognitive outcome after aneurysmal subarachnoid hemorrhage: time course of recovery and relationship to clinical, radiological, and management parameters. Neurosurgery.

[29] Frazer D, Ahuja A, Watkins L, Cipolotti L (2007). Coiling versus clipping for the treatment of aneurysmal subarachnoid hemorrhage: a longitudinal investigation into cognitive outcome. Neurosurgery.

[30] de Oliveira Manoel AL, Mansur A, Murphy A, Turkel-Parrella D, Macdonald M, Macdonald RL, Montanera W, Marotta TR, Bharatha A, Effendi K, Schweizer TA (2014). Aneurysmal subarachnoid haemorrhage from a neuroimaging perspective. Crit Care.

[31] Rowland MJ, Garry P, Ezra M, Corkill R, Baker I, Jezzard P, Westbrook J, Douaud G, Pattinson KTS (2021). Early brain injury and cognitive impairment after aneurysmal subarachnoid haemorrhage. Sci Rep.

[32] Alexander AL, Lee JE, Lazar M, Field AS (2007). Diffusion tensor imaging of the brain. Neurotherapeutics.

[33] Reijmer YD, van den Heerik MS, Heinen R, Leemans A, Hendrikse J, de Vis JB, van der Kleij LA, Lucci C, Hendriks ME, van Zandvoort MJ, Huenges Wajer IM, Visser-Meily JA, Rinkel GJ, Biessels GJ, Vergouwen MD (2018). Microstructural white matter abnormalities and cognitive impairment after aneurysmal subarachnoid hemorrhage. Stroke.

[34] Fragata I, Canhão Patrícia (2019). Imaging predictors of outcome in acute spontaneous subarachnoid hemorrhage: a review of the literature. Acta Radiol.

[35] Jensen JH, Helpern JA, Ramani A, Lu H, Kaczynski K (2005). Diffusional kurtosis imaging: the quantification of non-gaussian water diffusion by means of magnetic resonance imaging. Magn Reson Med.

[36] Weber RA, Hui ES, Jensen JH, Nie X, Falangola MF, Helpern JA, Adkins DL (2015). Diffusional kurtosis and diffusion tensor imaging reveal different time-sensitive stroke-induced microstructural changes. Stroke.

[37] Raab P, Hattingen E, Franz K, Zanella FE, Lanfermann H (2010). Cerebral gliomas: diffusional kurtosis imaging analysis of microstructural differences. Radiology.

[38] Abdel Razek AAK, Talaat M, El-Serougy L, Gaballa G, Abdelsalam M (2019). Clinical applications of arterial spin labeling in brain tumors. J Comput Assist Tomogr.

[39] Romano DG, Frauenfelder G, Locatelli G, Panza MP, Siani A, Tartaglione S, Leonini S, Beomonte Zobel B, Saponiero R (2019). Arterial spin labeling magnetic resonance imaging to diagnose contrast-induced vasospasm after intracranial stent embolization. World Neurosurg.

[40] Johnson NA, Jahng G, Weiner MW, Miller BL, Chui HC, Jagust WJ, Gorno-Tempini ML, Schuff N (2005). Pattern of cerebral hypoperfusion in Alzheimer disease and mild cognitive impairment measured with arterial spin-labeling MR imaging: initial experience. Radiology.

[41] Sati P, Patil S, Krueger G, Derbyshire JA, Reich DS (2019). Fast sub-millimeter whole-brain magnetic resonance imaging of novel biomarkers for multiple sclerosis.

[42] Krishnan A, Lansley J, Jäger H Rolf, Mankad K (2015). New vistas in clinical practice: susceptibility-weighted imaging. Quant Imaging Med Surg.

[43] Wu Z, Li S, Lei J, An D, Haacke E (2010). Evaluation of traumatic subarachnoid hemorrhage using susceptibility-weighted imaging. Am J Neuroradiol.

[44] Sun H, Klahr AC, Kate M, Gioia LC, Emery DJ, Butcher KS, Wilman AH (2018). Quantitative susceptibility mapping for following intracranial hemorrhage. Radiology.

[45] Deistung A, Schweser F, Reichenbach JR (2017). Overview of quantitative susceptibility mapping. NMR Biomed.

[46] Emblem KE, Mouridsen K, Bjornerud A, Farrar CT, Jennings D, Borra RJH, Wen PY, Ivy P, Batchelor TT, Rosen BR, Jain RK, Sorensen AG (2013). Vessel architectural imaging identifies cancer patient responders to anti-angiogenic therapy. Nat Med.

[47] Zhang K, Yun SD, Triphan SMF, Sturm VJ, Buschle LR, Hahn A, Heiland S, Bendszus M, Schlemmer H, Shah NJ, Ziener CH, Kurz FT (2019). Vessel architecture imaging using multiband gradient-echo/spin-echo EPI. PLoS One.

[48] Jespersen SN, Østergaard Leif (2012). The roles of cerebral blood flow, capillary transit time heterogeneity, and oxygen tension in brain oxygenation and metabolism. J Cereb Blood Flow Metab.

[49] Kang KM, Choi SH, Hwang M, Yoo R, Yun TJ, Kim J, Sohn C (2018). Application of synthetic MRI for direct measurement of magnetic resonance relaxation time and tumor volume at multiple time points after contrast administration: preliminary results in patients with brain metastasis. Korean J Radiol.

[50] Teasdale GM, Drake CG, Hunt W, Kassell N, Sano K, Pertuiset B, De Villiers JC (1988). A universal subarachnoid hemorrhage scale: report of a committee of the World Federation of Neurosurgical Societies. J Neurol Neurosurg Psychiatry.

[51] Cardol M, de Haan RJ, de Jong BA, van den Bos GA, de Groot IJ, Tate RL (2010). Impact on Participation and Autonomy (IPA) Questionnaire. A Compendium of Tests, Scales and Questionnaires.

[52] Jenkinson M, Beckmann CF, Behrens TE, Woolrich MW, Smith SM (2012). FSL. Neuroimage.

[53] Peduzzi P, Concato J, Kemper E, Holford TR, Feinstein AR (1996). A simulation study of the number of events per variable in logistic regression analysis. J Clin Epidemiol.

[54] Vittinghoff E, McCulloch CE (2007). Relaxing the rule of ten events per variable in logistic and Cox regression. Am J Epidemiol.

[55] de Bresser J, Vincken KL, Kaspers AJ, Rinkel GJ, Viergever MA, Biessels GJ (2012). Quantification of cerebral volumes on MRI 6 months after aneurysmal subarachnoid hemorrhage. Stroke.

[56] Bendel P, Koivisto T, Niskanen E, Könönen Mervi, Aikiä Marja, Hänninen Tuomo, Koskenkorva P, Vanninen R (2009). Brain atrophy and neuropsychological outcome after treatment of ruptured anterior cerebral artery aneurysms: a voxel-based morphometric study. Neuroradiology.

[57] Macdonald RL, Jaja B, Cusimano MD, Etminan N, Hanggi D, Hasan D, Ilodigwe D, Lantigua H, Le Roux P, Lo B, Louffat-Olivares A, Mayer S, Molyneux A, Quinn A, Schweizer TA, Schenk T, Spears J, Todd M, Torner J, Vergouwen MDI, Wong GKC, Singh J, SAHIT Collaboration (2013). SAHIT Investigators--on the outcome of some subarachnoid hemorrhage clinical trials. Transl Stroke Res.

[58] Geraghty JR, Lara-Angulo MN, Spegar M, Reeh J, Testai FD (2020). Severe cognitive impairment in aneurysmal subarachnoid hemorrhage: Predictors and relationship to functional outcome. J Stroke Cerebrovasc Dis.

[59] Quinn AC, Bhargava D, Al-Tamimi YZ, Clark MJ, Ross SA, Tennant A (2014). Self-perceived health status following aneurysmal subarachnoid haemorrhage: a cohort study. BMJ Open.

[60] Nesvick CL, Oushy S, Rinaldo L, Wijdicks EF, Lanzino G, Rabinstein AA (2019). Clinical complications and outcomes of angiographically negative subarachnoid hemorrhage. Neurology.

